# Impaired Clinical Efficacy of Aspirin in Hypoalbuminemic Patients With Diabetes Mellitus

**DOI:** 10.3389/fphar.2021.695961

**Published:** 2021-06-22

**Authors:** Angela Sciacqua, Francesco Andreozzi, Elena Succurro, Daniele Pastori, Vittoria Cammisotto, Giuseppe Armentaro, Gaia C. Mannino, Teresa Vanessa Fiorentino, Pasquale Pignatelli, Dominick J. Angiolillo, Giorgio Sesti, Francesco Violi

**Affiliations:** ^1^ Department of Medical and Surgical Sciences, University Magna Graecia of Catanzaro, Catanzaro, Italy; ^2^ Department of Clinical, Internal, Anaesthesiological, and Cardiovascular Sciences, Sapienza University of Rome, Rome, Italy; ^3^ Department of General Surgery and Surgical Specialty Paride Stefanini, Sapienza University of Rome, Rome, Italy; ^4^ Mediterranea Cardiocentro, Napoli, Italy; ^5^ Division of Cardiology, University of Florida College of Medicine, Jacksonville, FL, United States; ^6^ Department of Clinical and Molecular Medicine, Sapienza, University of Rome, Rome, Italy

**Keywords:** diabetes, albumin, thromboxane, platelet, aspirin, cardiovascular events

## Abstract

**Objective:** To investigate the impact of albumin levels on the aspirin efficacy, since aspirin inhibits platelet aggregation (PA) by cyclooxygenase one irreversible acetylation that is less effective in patients with type 2 diabetes mellitus (T2DM).

**Patients and Methods:** A total of 612 aspirin (100 mg/day)-treated T2DM patients were followed-up for 54.4 ± 7.3 months. The primary endpoint, a composite of cardiovascular events (CVEs) including CV death, myocardial infarction, ischemic stroke and coronary revascularization, was analysed according to baseline values of serum albumin (≥ or < 3.5 g/dL). Serum thromboxane (Tx)B_2_ was also measured.

**Results:** 250 (40.8%) patients had serum albumin < 3.5 g/dL; these patients were overweight and had higher values of fibrinogen (*p* = 0.009), high sensitivity C-reactive protein (*p* = 0.001) and fasting plasma glucose (*p* < 0.0001) compared to those with albumin ≥ 3.5 g/dL. During follow-up, 86 CVEs were recorded, 49 and 37 in patients with serum albumin < or ≥3.5 g/dL, respectively (*p* = 0.001). At multivariable Cox regression analysis, serum albumin < 3.5 g/dL (hazard ratio [HR] 1.887, 95% confidence interval [CI] 1.136–3.135, *p* = 0.014), age (HR 1.552 for every 10 years, 95%CI 1.157–2.081, *p* = 0.003), fasting plasma glucose (HR 1.063, 95%CI 1.022–1.105, *p* = 0.002) and beta-blocker use (HR 0.440, 95%CI 0.270–0.717, *p* = 0.001) were associated to CVEs. Serum TxB_2_ levels (*n* = 377) were 0.32 ± 0.12 and 0.24 ± 0.12 ng/ml in patients with albumin < or ≥ 3.5 g/dL, respectively (*p* < 0.001).

**Conclusion:** In T2DM patients, the efficacy of aspirin varies according to albumin levels. Hypoalbuminemia associated with impaired TxB_2_ inhibition and an increased risk of long-term CVEs.

## Introduction

Patients with type II diabetes mellitus (T2DM) are at increased risk of atherosclerotic disease and its thrombotic complications (Wright et al., 2020). Several mechanisms contribute to the increased atherothrombotic disease manifestations, such as myocardial infarction and stroke, in patients with T2DM, including their pro-thrombotic status consequent to activation of platelets (Morel et al., 2013).

These observations underscore the importance of antiplatelet therapies to mitigate such risk. Low-dose aspirin (81–100 mg/day), which irreversibly inhibits cyclooxygenase (COX)-1 and preventing the biosynthesis of thromboxane B_2_ (TxB_2_), is the most used antiplatelet agent to prevent acute cardiovascular events (Mora and Manson, 2016). Studies conducted in high-risk patients with established atherosclerotic disease have consistently shown that aspirin treatment is associated with a reduced risk of cardiovascular events, albeit at the expense of an increased risk of bleeding (Zheng and Roddick, 2019).

However, the benefit of aspirin is less evident for primary prevention in T2DM patients (Bowman et al., 2018; Capodanno and Angiolillo, 2020; Angiolillo and Capodanno, 2021). Impaired COX-1 inhibition, also referred to as “aspirin resistance”, is detectable in aspirin-treated patients with T2DM and associated with worse outcomes (Eikelboom et al., 2008). However, the mechanisms underlying such “aspirin resistance” in patients with T2DM have not been fully elucidated, and it is not clear if it depends on factors intrinsic or extrinsic to the platelet. Indeed, accelerated platelet turnover rates in T2DM which would hamper the benefits of aspirin, characterized by limited circulating half-life, when given once daily, may have a contributing role (Santilli et al., 2015; Santos-Gallego and Badimon, 2021). These observations have suggested that twice daily administration of aspirin may lead to more effective platelet inhibitory effects, although this has not been tested clinically (Capodanno et al., 2011; Santos-Gallego and Badimon, 2021). However, other factors may also be involved.

Albumin is an acute phase reactive protein with antioxidant and antiplatelet activity *in vitro* (Belinskaia et al., 2020). We have previously reported that albumin dose-dependently inhibits platelet aggregation with a Nox2-related oxidative stress mechanism *in vitro* and that its supplementation in human impairs platelet aggregation (Basili et al., 2019). Also, previous studies reported that serum albumin <3.5 g/dL is associated with an increased risk of arterial and venous thrombosis (Chi et al., 2019; Pignatelli et al., 2020; Ronit et al., 2020) but the biological plausibility of this association as well as its impact with the clinical efficacy of antiplatelet drugs such as aspirin has not been elucidated. Based on these observations, we assessed if COX-1 inhibition mediated by aspirin could be impaired in the presence of hypoalbuminemia resulting in lower clinical efficacy. We performed an observational cohort study to assess the relationship between albumin serum levels and cardiovascular events in aspirin-treated T2DM patients.

## Methods

### Study Population

From the entire study cohort of the CATAnzaro Metabolic RIsk factors (CATAMERI) Study, an ongoing longitudinal observational study assessing cardio-metabolic risk in individuals, recruited at the University Hospital of Catanzaro. Recruitment mechanisms include word-of-mouth, fliers, and newspaper advertisements. All subjects were consecutively recruited according to the following inclusion criteria: age >18 and positivity for one or more cardio-metabolic risk factors including family history of diabetes, dysglycemia, hypertension, dyslipidemia, and overweight/obesity (Andreozzi et al., 2007). Patients recruited between January 2006 and December 2014 (*n* = 1,658) completed a 5-years follow-up visit. Of these, 612 patients had T2DM, without a previous cardiovascular event, on treatment with low-dose-aspirin and with available serum albumin levels.

All patients underwent an assessment of their past medical history to assess the presence of cardiovascular risk factors and medical treatment. Moreover, complete anthropometrical assessment and measurement of height, weight, and body mass index (BMI) was performed.

At baseline, subjects were excluded if they had previous diagnosis of type 1 diabetes, a history of cardiovascular disease, chronic gastrointestinal or autoimmune diseases, history of any malignant disease and alcohol or drug abuse, liver, or kidney failure. At baseline all subjects had an estimated glomerular filtration rate (e-GFR) > 60 ml/min/1.73 m^2^ and none had a diagnosis of nephrotic syndrome.

T2DM was defined according to the American Diabetes Association (ADA) criteria: HbA1c ≥ 6.5% (48 mmol/mol), fasting plasma glucose (FPG) ≥126 mg/dL (7 mmol/L), 2-h post-load glucose ≥200 mg/dL (11.1 mmol/L) or use of glucose-lowering medications (American Diabetes Associa, 2015).

The study was approved by the local Institutional Ethics Committees of University “Magna Graecia” of Catanzaro. Written informed consent was obtained from each subject in accordance with principles of the Declaration of Helsinki.

### Laboratory Determinations

All laboratory measurements were performed after at least 12 fasting hours. Serum albumin was measured with a colorimetric spectrophotometric method (Bromocresol green), ALT and AST by pyridoxal phosphate activated (liquid reagent) and γ-GT was evaluated by standardized against Szasz (COBAS Integra 800—Roche Diagnostics GmbH, Mannheim, Germany).

Plasma glucose was determined by the glucose oxidase method (Glucose analyzer, Beckman Coulter, Milan). Triglycerides, low and high-density lipoprotein (LDL, HDL) and cholesterol concentrations were measured by enzymatic methods (Roche Diagnostics GmbH, Mannheim, Germany). Plasma insulin was determined by a chemiluminescence-based assay (Immulite^®^, Siemens Healthcare, Italy).

Insulin Resistance was estimated by calculation of homeostasis model assessment (HOMA) index, derived from the fasting glucose and insulin concentrations according to the formula: HOMA = [insulin (μU/mL) × glucose (mmol/L)]/22.5.

High sensitivity C reactive protein (hsCRP) levels were measured in plasma samples with an automated instrument by a quantitative method through the use of mouse monoclonal antibodies (CardioPhase^®^ hsCRP, Siemens Healthcare, Italy).

Serum creatinine was evaluated by the Roche Creatinine Plus assay (Ho_man-La Roche, Basel, Switzerland) on a clinical chemistry analyser (Roche/Hitachi Modular Analytics System, P Module). Renal function, assessed by e-GFR, was calculated according to the equation suggested by the Chronic Kidney Disease Epidemiology (CKD-EPI) Collaboration group (Levey et al., 2009). Serum uric acid was measured by the URICASE/POD method on an automated analyser (Boehringer Mannheim, Mannheim, Germany).

### Serum Thromboxane

Blood samples to measure biomarkers of platelet activation were obtained within 24 h of admission. Samples were taken from patients who had fasted for at least 12 h. Serum was separated by centrifugation and frozen at −80°C until use. Serum TxB_2_ was measured using an enzyme-linked immunosorbent assay commercial kit (R and D Systems, Inc., Minneapolis, Minnesota) and expressed as nanograms per millilitre. Intra- and inter-assay coefficients of variation for the TxB_2_ assay kit were 5.9 and 8.9%, respectively.

### Follow-Up and Cardiovascular Events

Clinical follow-up was performed by outpatient clinical visits or hospitalization and by a telephone questionnaire. Complete data were available for all participants. At the time of the follow-up visit, none of the patients reported a history of bleeding. Regarding clinical events a validation by source data (hospital records, death certificates or other original documents) was required. Fatal and non-fatal acute myocardial infarction (AMI) and stroke, unstable angina, coronary revascularization procedures performed not in emergency conditions (percutaneous interventions and bypass graft surgery), cardiovascular death or death for any cause were considered as clinical events. Adjudication of events was performed by three investigators (FA, ES, and AS) who were unaware of baseline serum albumin. AMI occurrence was defined according to the criteria of the European Society of Cardiology/American College of Cardiology Foundation/American Heart Association/World Heart Federation (Thygesen et al., 2007). Stroke diagnosis was based on the sudden onset of a new neurological deficit persisting for at least 24 h and confirmed by radiological findings (Adams et al., 2007). The primary endpoint was defined as the occurrence of cardiovascular events (CVEs), including cardiovascular death, AMI, ischemic stroke, and coronary revascularization procedure.

### Statistical Analysis

Unpaired Student’ t-test was utilized to test the differences between clinical and biological data when expressed as continuous variables and the χ^2^ test was considered for categorical variables. Data are expressed as mean ± standard deviation (SD) or as percent frequency. Event rate is reported as the number of events per 100 patient-year, in this context the date of censorship was defined as the first clinical event for patients who experienced multiple events and as the last contact for patients without. Survival curves according to serum albumin levels cut-off value were estimated by using the Kaplan–Meier product-limit method and compared by using the Mantel (logistic rank) test.

The effect of several prognostic factors on CVEs was evaluated by using a Cox regression model, considering the following variables: serum albumin as dichotomous value (<3.5 or ≥3.5 g/dL), age, BMI, HDL-cholesterol, hs-CRP, sex, smoking, FPG, fibrinogen, diabetes duration and the different pharmacological treatments. The multiple Cox regression model was built by considering all variables significantly associated with the incident risk of CVEs at univariable Cox regression analysis. We provided a Cox model of adequate statistical power (at least 10 events for each variable included in the final model). Data are expressed as hazard ratio (HR) and *p* value. In two-tailed tests, a value of *p* < 0.05 was considered statistically significant. The statistical analysis was performed using the statistical package SPSS 20.0 for Windows (SPSS Inc., Chicago, Illinois, United States).

## Results

The mean age of the overall study population was 60.4 ± 10.3 years and 44.4% were women (Table 1). The mean duration of diabetes was 5.1 ± 1.3 years**.** Mean serum albumin was 4.0 ± 0.8 g/dL. Serum levels of albumin <3.5 g/dL was encountered in 250 (41%) patients. Table 1 reports clinical characteristics of the study cohort according to serum albumin levels (<or ≥3.5 g/dL). Patients with low serum albumin were more frequently obese (*p* < 0.0001), with lower HDL cholesterol (*p* = 0.043), higher fibrinogen (*p* = 0.009) and hs-CRP (*p* = 0.001) (Table 1). No differences between the two groups were observed in metabolic control, as assessed by HbA1c levels, HOMA insulin resistance index, eGFR or liver enzymes. Overall, nearly 70% of patients were treated with anti-hypertensive drugs, 58.5% were on oral antidiabetic drugs, 17% on insulin and the remaining 24.5% of the diabetic patients were on diet therapy. Of interest, even though enrolled patients showed a high CV risk, only 54% were on statin therapy at baseline. However, there were no differences in treatment with statin in the two groups in study (Table 2). Diabetic patients in study were on treatment only with metformin or sulfonylureas and there were no differences in distribution of oral hypoglycemic drugs or cardioprotective drugs in the two groups (Table 2).

**TABLE 1:** Baseline characteristics of the study population according to serum albumin levels.

Variables	All (*n* = 612)	Albumin <3.5 g/dl (*n* = 250)	Albumin ≥3.5 g/dl (*n* = 362)	*p*
Albumin**,** *g/dl*	4.0 ± 0.8	3.2 ± 0.2	4.6 ± 0.4	<0.0001
Sex, *m/f*	340/272	140/110	200/162	0.854
Age, *years*	60.4 ± 10.3	60.4 ± 9.1	60.3 ± 11.1	0.880
Smokers*,n (%)*	97 (15.4)	31 (12.4)	66 (18.2)	0.052
SBP, *mmHg*	138.9 ± 20.6	139.4 ± 20.9	138.6 ± 20.4	0.648
DBP, *mmHg*	80.1 ± 11.8	80.1 ± 12.2	80.2 ± 11.6	0.845
Hypertension, *n (%)*	486 (79.4)	199 (79.6)	287 (79.3)	0.923
BMI, *Kg/m* ^*2*^	31.7 ± 6.7	33.1 ± 7.9	30.7 ± 5.6	<0.0001
HDL cholesterol, *mg/dl*	45.7 ± 13.7	44.4 ± 13.6	46.6 ± 13.6	0.043
LDL cholesterol, *mg/dl*	112.6 ± 39.8	109.9 ± 39.1	114.4 ± 40.3	0.184
Triglyceride, *mg/dl*	156.7 ± 90.1	156.9 ± 75.6	156.6 ± 98.8	0.965
HbA1c, *%*	7.3 ± 1.4	7.4 ± 1.3	7.3 ± 1.4	0.428
Uric acid, *mg/dl*	5.6 ± 1.5	5.6 ± 1.7	5.6 ± 1.4	0.886
e-GFR, *ml/min/m* ^*2*^	101.2 ± 36.4	99.3 ± 39.1	102.5 ± 34.4	0.309
Fibrinogen, *mg/dl*	344.7 ± 92.9	357.6 ± 105.4	335.4 ± 81.5	0.009
Hs-CRP, *mg/dl*	4.8 ± 4.0	5.5 ± 4.0	4.3 ± 4.0	0.001
WBC, *cells x μl*	7525.0 ± 2123.8	7581.8 ± 2302.5	7485.7 ± 1993.2	0.595
PLT, *x10* Mora and Manson, (2016) */µL*	240.2 ± 70.3	242.2 ± 79.7	238.7 ± 63.0	0.566
AST, *UI/L*	23.6 ± 14.1	23.1 ± 16.8	23.9 ± 12.1	0.482
ALT, *UI/L*	28.5 ± 21.3	26.7 ± 22.2	29.8 ± 20.6	0.080
γ-GT, *UI/L*	36.7 ± 31.1	37.4 ± 28.9	36.2 ± 32.6	0.643
FPG, *mg/dl*	160.8 ± 58.2	177.3 ± 66.6	149.3 ± 48.4	<0.0001
FPI, *µU/ml*	18.0 ± 14.4	18.6 ± 15.6	17.7 ± 13.7	0.512
HOMA	6.7 ± 6.2	7.3 ± 6.3	6.3 ± 6.1	0.101
Diabetes duration	5.1 ± 1.3	5.1 ± 1.4	5.2 ± 1.2	0.873

SBP, systolic blood pressure; DBP, diastolic blood pressure; BMI, body mass index; HDL, high density lipoproteins; LDL, low density lipoproteins; HbA1c, glycated haemoglobin; e-GFR, estimated glomerular filtration rate; hs-CRP, high sensitivity C reactive protein; WBC, white blood cells; PLT, platelets; AST, aspartate aminotransferase; ALT, alanine aminotransferase; gGT, gamma-glutamyltransferase; FPG, fasting plasma glucose; FPI, fasting plasma insulin; HOMA, homeostatic model assessment.

**TABLE 2:** Drug treatment of the study population according to serum albumin levels.

	All (*n* = 612)	Albumin < 3.5 g/dL (*n* = 250)	Albumin ≥ 3.5 g/dL (*n* = 362)	*p* value
RAS inhibitors, *n (%)*	427 (69.8)	175 (70)	252 (69.6)	0.918
Calcium channel blockers, *n (%)*	148 (24.2)	60 (24)	88 (24.3)	0.930
Beta-blockers, *n (%)*	212 (34.6)	87 (34.8)	125 (34.5)	0.945
Diuretics, *n (%)*	202 (33.0)	82 (32.8)	120 (33.1)	0.928
Other Antihypertensive drugs*, n (%)*	46 (7.5)	19 (7.6)	27 (7.4)	0.948
Statins, *n (%)*	331 (54.1)	135 (54)	196 (54.1)	0.962
Insulin therapy, *n (%)*	104 (17.0)	42 (16.8)	62 (17.1)	0.915
OAD, *n (%)*	358 (58.5)	145 (58)	213 (58.8)	0.835

RAS, renin-angiotensin system; OAD, oral antidiabetic drugs.

### Low Serum Albumin and CVEs

**FIGURE 1:**
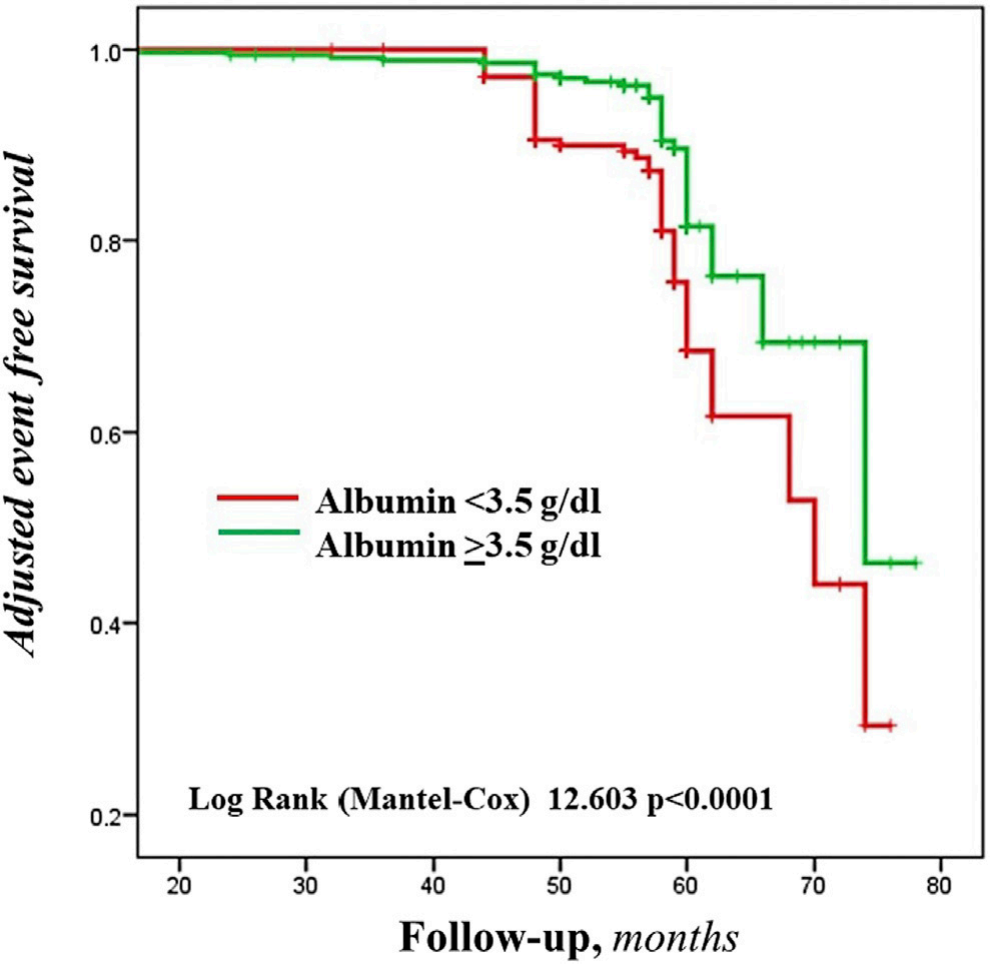
Adjusted Kaplan-Meier estimates of the cardiovascular events, according to the cut-off value of serum albumin.

During a mean follow-up of 54.4 months, 86 CVEs were recorded (incidence rate 3.10%/100 patient-year): 49 and 37 in the group of patients with serum albumin < and ≥3.5 g/dL, respectively (Log Rank test *p* < 0.0001, Figure 1). Table 3 reports the number and type of event in each study group.

**TABLE 3:** Clinical events in the study population according to serum albumin levels.

	Number of patients (number of events per 100 patient-year)			
	All (*n* = 612)	Albumin <3.5 g/dL (*n* = 250)	Albumin ≥3.5 g/dL (*n* = 362)	*p* value
Total mortality, *n (%)*	10 (0.36)	6 (0.54)	4 (0.24)	0.214
CVEs, *n (%)*	86 (3.10)	49 (4.39)	37 (2.24)	0.001
Cardiovascular mortality, *n (%)*	7 (0.25)	4 (0.36)	3 (0.18)	0.377
Nonfatal cardiovascular events, *n (%)*	79 (2.85)	45 (4.02)	34 (2.05)	0.001
Coronary, *n (%)*	65 (2.34)	37 (3.31)	28 (1.69)	0.005
Cerebrovascular, *n (%)*	14 (0.50)	8 (0.72)	6 (0.36)	0.209
Follow-up, *months*	54.4 ± 7.3	53.7 ± 7.2	54.8 ± 7.4	0.067

CVEs, cardiovascular events.

The incidence rates of CVEs (4.39 vs 2.24%/100 patient-year) and of coronary events (fatal/non-fatal AMI and coronary revascularization) (3.31 vs 1.69%/100 patient-year) were nearly doubled in patients with low serum albumin (Table 3).

Mean value of serum albumin was 3.8 ± 0.8 g/dL in patients with and 4.1 ± 0.7 g/dL in patients without CVEs during follow-up (*p* = 0.011). At univariable (Table 4, Panel A) and multivariable (Table 4, Panel B) Cox regression analysis, low serum albumin was associated with CVEs (HR 1.887, *p* = 0.014), along with age (HR 1.552 for every 10 years, *p* = 0.003), FPG (HR 1.063, *p* = 0.002) and beta-blockers (HR 0.440, *p* = 0.001).

**TABLE 4:** Univariable (panel A) and multivariable (panel B) Cox regression analysis for cardiovascular event occurrence (albumin as dichotomous variable).

		Hazard ratio	95%CI	*p*
Panel A				
Beta-blockers	Yes/no	0.507	0.294–0.874	0.015
Age	10 years	1.504	1.092–2.071	0.012
FPG	10 mg/dL	1.054	1.013–1.098	0.009
Albumin	<3.5 or ≥3.5 g/dL	1.820	1.081–3.064	0.024
Panel B				
Albumin	<3.5 or ≥3.5 g/dL	1.887	1.136–3.135	0.014
Age	10 years	1.552	1.157–2.081	0.003
FPG	10 mg/dl	1.063	1.022–1.105	0.002
Beta-blockers	Yes/no	0.440	0.270–0.717	0.001

Variables included in the model: age, BMI, HDL, hs-CRP, sex, smoking, FPG, albumin, RAS inhibitors, Calcium channel blockers, Beta-blockers, diuretics, statins, insulin therapy, oral antidiabetic drugs.

### Serum Thromboxane

The relationship between platelet biosynthesis of TxB_2_ and albumin levels was measured in 377 out of 612 patients. There were no differences in baseline characteristics compared with the entire cohort (data not shown). Mean serum TxB_2_ levels were 0.27 ± 0.12 ng/ml. Serum TxB_2_ was 0.32 ± 0.12 and 0.24 ± 0.12 ng/ml in patients with albumin < or ≥3.5 g/dL, respectively (*p* < 0.001). Serum albumin was inversely associated with serum TxB_2_ values above the median (univariable odds ratio 0.52, 95%CI 0.39–0.69, *p* < 0.001).

## Discussion

The present investigation aimed at assessing the impact of serum albumin levels on long-term outcomes in a cohort of aspirin-treated T2DM subjects without overt cardiovascular disease, showing hypoalbuminemia to be associated with an enhanced risk of cardiovascular events. Furthermore, we found impaired COX-1 inhibition in patients with low serum albumin, as shown by increased serum levels of TxB_2_ despite aspirin treatment. Interesting, our study showed a prevalence of hypoalbuminemia as high as 41% using a cut-off of 3.5 g/dL in our cohort of T2DM patients. An even higher prevalence, i.e,. 70.4%, has been previously reported in 280 patients affected by T2DM using <40 g/L as cut-off to define hypoalbuminemia (Marlow et al., 2011). The reason for hypoalbuminemia in T2DM may be multiple including loss of albumin for concomitant kidney disease, liver disease or infections (Gupta and Lis, 2010; Lyons et al., 2010). As albumin is an acute reactant protein, it is usually reduced during infections. According with this, respiratory infections activate platelets, increase TxB2, and increase the myocardial infarction risk (Santos-Gallego and Badimon, 2014); however, patients with acute infectious disease were excluded by the present study. Patients with T2DM are also characterized by chronic inflammation (Shoelson et al., 2006) and lower albumin is likely to be an epiphenomenon of inflammatory status as suggested by the significant increase of hs-CRP and fibrinogen in patients with serum albumin <3.5 g/dL. Kidney and hepatic diseases seem to have a minor role as no relationship was detected between hypoalbuminemia, glomerular filtration rate and transaminases. However, further studies are necessary to assess if macro/micro albuminuria, not assessed in the present study, has also a contributing role.

During a follow-up of approximately 54 months, 86 patients experienced CVEs resulting in a 3.1% annualized event rate. However, in patients with hypoalbuminemia the incidence of CVEs was approximately 2-fold higher (4.39%/100 patient-year) compared to those with normal albumin levels (2.24%/100 patient-year). The difference in incidence of CVEs was driven by coronary events while total mortality and stroke were not different between the two groups. The close association between serum albumin and CVEs was supported by the multivariable Cox proportional hazards regression analysis which confirmed an independent association between low serum albumin and CVEs.

**FIGURE 2:**
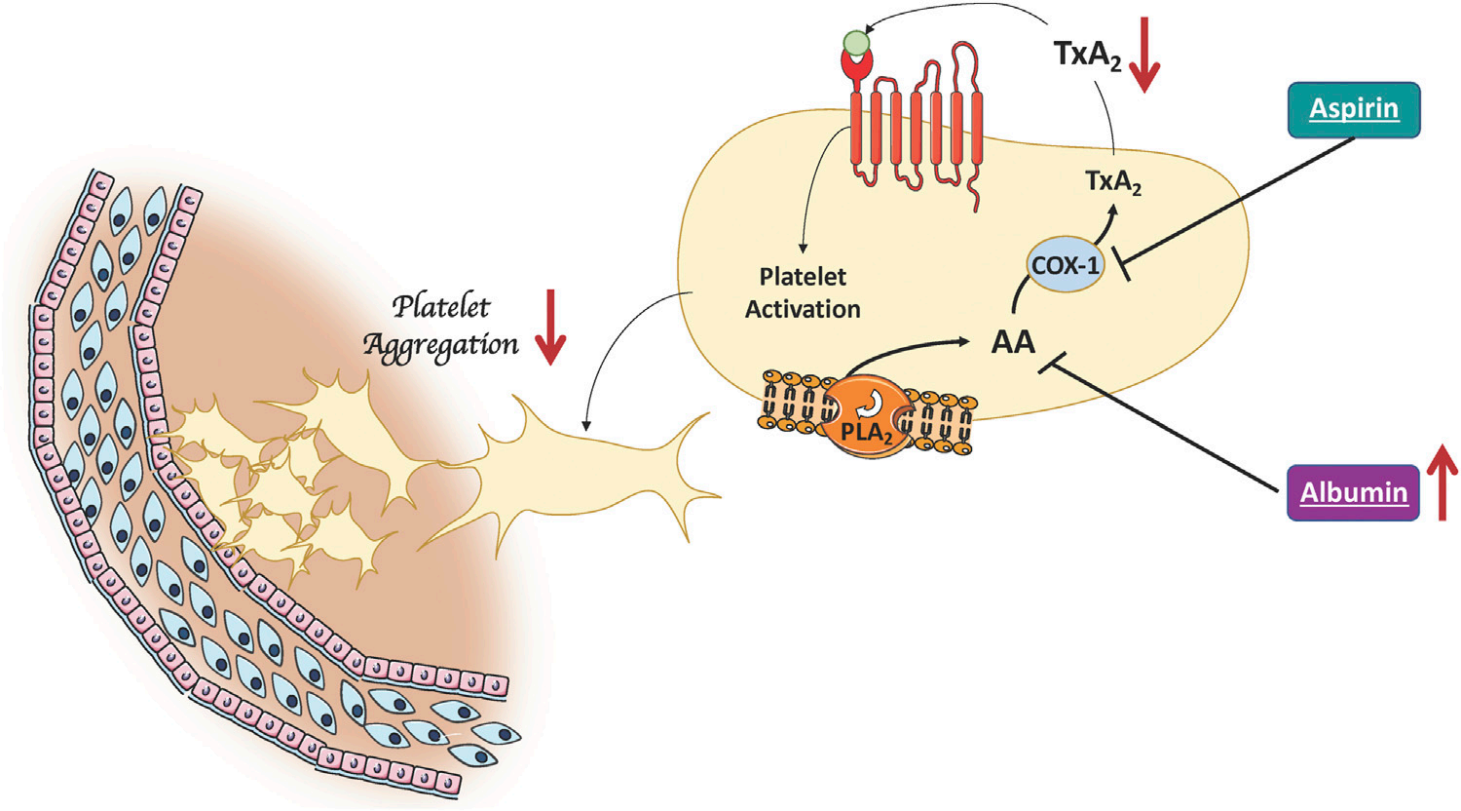
Albumin lowers platelet aggregation. High albumin levels improve the clinical efficacy of aspirin by binding arachidonic acid released by PLA2-activation. This results in a decreased of platelet TxA2 production which in turn is no longer available for platelet activation (AA, Arachidonic Acid; COX-1, cyclooxygenase-1; PLA_2_, Phospholipase A2; TxA_2_, Thromboxane A2).

Findings from the present study add to previous evidence showing that platelet resistance to aspirin in T2DM is dependent on several factors including the variable recovery of platelet cyclooxygenase activity (Rocca et al., 2012) and oxidative stress-mediated platelet isoprostane overproduction (Cangemi et al., 2012). However, the clinical impact of these functional changes is still unknown. Indeed, our observation that in aspirin-treated patients the coexistence of low albumin levels significantly impairs the clinical efficacy of aspirin, suggests a new mechanism potentially contributing to the aspirin resistance in patients with T2DM (Figure 2). We focused on albumin as previous studies showed that albumin binds arachidonic acid released by PLA_2_-activation, which, in turn is no longer available for TxA_2_ biosynthesis or directly binding TxA_2_ (Maclouf et al., 1980; Porcellati et al., 1995). Our group supported and extended these reports by showing that albumin dose-dependently lowers platelet aggregation with a mechanism related to its antioxidant effect; this finding was substantiated by an interventional study in patients with low serum levels of albumin in whom intravenous administration of albumin significantly reduced platelet aggregation (Basili et al., 2019).

To further address this issue, we analysed serum levels of TxB_2_, which reflects platelet biosynthesis of TxA_2_, in samples taken from a sub-group of the entire cohort having serum albumin < or ≥3.5 g/dL. In this cohort, serum levels of TxB_2_ were 0.27 ng/ml which is consistent with serum TxB_2_ levels detected in a population of patients with T2DM on chronic treatment with aspirin (Rocca et al., 2012). However, significantly higher levels of serum TxB_2_ were detected in patients with serum albumin <3.5 g/dL compared with those having albumin ≥3.5 g/dL, suggesting impaired COX-1 inhibition in hypo-albuminemic T2DM aspirin-treated patients. A previous study showed an inverse relationship between platelet activation and serum albumin <3.5 g/dL in patients affected by stable coronary artery disease but data regarding COX-1 inhibition as well as its impact with clinical outcomes was not investigated (Shiyovich et al., 2020).

We cannot also exclude that the efficacy of aspirin may be modified by the serum levels of albumin. Thus, albumin binds many circulating drugs, including non-steroidal anti-inflammatory drugs, potentially modulating their efficacy (Koch-Weser and Sellers, 1976). However, a previous study showed that aspirin efficacy is not modified by protein concentration (Warner et al., 2006). In addition, interactions with strong competitors for albumin binding may also affect aspirin efficacy.

### Study Limitation and Implication

The present investigation did not assess the mechanisms of reduced levels of serum albumin, which are likely to be multifactorial in T2DM. The inverse association of albumin with hs-CRP is consistent with the anti-inflammatory properties of albumin and its consumption with chronic inflammation, that may complicate the clinical course of diabetes. Loss of albumin from the kidney or detrimental changes of nutritional status can also contribute to low serum albumin and should be investigated. Our study measured serum albumin levels only at baseline and did not assess whether increasing albumin serum levels could result in an improved response to aspirin and eventually CVEs lowering; this issue deserves further investigations. Compliance to aspirin and other pharmacological treatment was assessed only by telephone contacts and scheduled clinical visits that patients made at the referral center as reported in own medical records. Despite platelet TxB_2_ reflects platelet activation, we did not measure other markers of platelet activation such as soluble P-selectin, which reflects *in vivo* platelet activation. Finally, the measurements of albumin levels at the beginning of the study and the lack of a control group are other limitations of the study. Even if our findings cannot be extrapolated to subjects without T2DM, analysis of serum levels of albumin may be proposed as novel tool to assess the antiplatelet activity of aspirin in patients with putative hypoalbuminemia.

## Conclusion

In conclusion, we provide evidence that a considerable proportion of patients with T2DM exhibits low serum levels of albumin. Importantly, patients with this laboratory feature display impaired COX-1 inhibition by aspirin and experience a higher rate of cardiovascular events. This study gives new insights to the understanding of the reduced effectiveness of aspirin in T2DM and provides a rationale for an interventional trial to assess if albumin supplementation may improve aspirin responsiveness and positively affect the risk of experiencing a future cardiovascular event.

## Data Availability

The raw data supporting the conclusions of this article will be made available by the authors, without undue reservation, to any qualified researcher.

## Abbreviations

ADA, American Diabetes Association; AMI, acute myocardial infarction; BMI, body mass index; CATAMERI, CATAnzaro Metabolic RIsk factors; CKD-EPI, Chronic Kidney Disease Epidemiology; COX, cyclooxygenase; CVEs, cardiovascular events; e-GFR, estimated glomerular filtration rate; FPG,fastingplasma glucose; HDL, high-density lipoprotein; HOMA, homeostasis model assessment; HR, hazard ratio; hsCRP, highsensitivity C reactive protein; LDL, low-density lipoprotein; NADPH, nicotinamide adenine dinucleotide phosphate; SD, standard deviation; T2DM, type II diabetes mellitus; TxB_2_, thromboxane B_2_.

## References

[B1] Adams H. P. Del Zoppo G. Alberts M. J. Bhatt D. L. Brass L. Furlan A. (2007). Guidelines for the Early Management of Adults with Ischemic Stroke. Stroke. 38 (5), 1655–1711. 10.1161/STROKEAHA.107.181486 17431204

[B2] American Diabetes Association (2015). (2) Classification and Diagnosis of Diabetes. Diabetes Care. 38, S8–S16. 10.2337/dc15-S005 25537714

[B3] Andreozzi F. Succurro E. Mancuso M. R. Perticone M. Sciacqua A. Perticone F. (2007). Metabolic and Cardiovascular Risk Factors in Subjects with Impaired Fasting Glucose: the 100versus 110 mg/dL Threshold. Diabetes Metab. Res. Rev. 23 (7), 547–550. 10.1002/dmrr.724 17311284

[B4] Angiolillo D. J. Capodanno D. (2021). Aspirin for Primary Prevention of Cardiovascular Disease in the 21st Century: A Review of the Evidence. Am. J. Cardiol. 144, S15–S22. 10.1016/j.amjcard.2020.12.022 33706985

[B5] Basili S. Carnevale R. Nocella C. Bartimoccia S. Raparelli V. Talerico G. (2019). Serum Albumin Is Inversely Associated with Portal Vein Thrombosis in Cirrhosis. Hepatol. Commun. 3 (4), 504–512. 10.1002/hep4.1317 30976741 PMC6442692

[B6] Belinskaia D. A. Voronina P. A. Shmurak V. I. Vovk M. A. Batalova A. A. Jenkins R. O. (2020). The Universal Soldier: Enzymatic and Non-enzymatic Antioxidant Functions of Serum Albumin. Antioxidants. 9 (10), 966–994. 10.3390/antiox9100966 PMC7601824 33050223

[B7] Bowman L. Bowman L. Mafham M. Wallendszus K. Stevens W. Buck G. ASCEND Study Collaborative Group (2018). Effects of Aspirin for Primary Prevention in Persons with Diabetes Mellitus. N. Engl. J. Med. 379 (16), 1529–1539. 10.1056/nejmoa1804988 30146931

[B8] Cangemi R. Pignatelli P. Carnevale R. Nigro C. Proietti M. Angelico F. (2012). Platelet Isoprostane Overproduction in Diabetic Patients Treated with Aspirin. Diabetes. 61 (6), 1626–1632. 10.2337/db11-1243 22427378 PMC3357260

[B9] Capodanno D. Angiolillo D. J. (2020). Antithrombotic Therapy for Atherosclerotic Cardiovascular Disease Risk Mitigation in Patients with Coronary Artery Disease and Diabetes Mellitus. Circulation. 142 (22), 2172–2188. 10.1161/CIRCULATIONAHA.120.045465 33253005

[B10] Capodanno D. PatelDharmashankar A. K. Dharmashankar K. Ferreiro J. L. Ueno M. Kodali M. (2011). Pharmacodynamic Effects of Different Aspirin Dosing Regimens in Type 2 Diabetes Mellitus Patients with Coronary Artery Disease. Circ. Cardiovasc. Interv. 4 (2), 180–187. 10.1161/CIRCINTERVENTIONS.110.960187 21386092

[B11] Chi G. Gibson C. M. Liu Y. Hernandez A. F. Hull R. D. Cohen A. T. (2019). Inverse Relationship of Serum Albumin to the Risk of Venous Thromboembolism Among Acutely Ill Hospitalized Patients: Analysis from the APEX Trial. Am. J. Hematol. 94 (1), 21–28. 10.1002/ajh.25296 30252149

[B12] Eikelboom J. W. Hankey G. J. Thom J. Bhatt D. L. Steg P. G. Montalescot G. (2008). Incomplete Inhibition of Thromboxane Biosynthesis by Acetylsalicylic Acid. Circulation. 118 (17), 1705–1712. 10.1161/CIRCULATIONAHA.108.768283 18838564

[B13] Gupta D. Lis C. G. (2010). Pretreatment Serum Albumin as a Predictor of Cancer Survival: A Systematic Review of the Epidemiological Literature. Nutr. J. 9 (1), 69. 10.1186/1475-2891-9-69 21176210 PMC3019132

[B14] Koch-Weser J. Sellers E. M. (1976). Binding of Drugs to Serum Albumin. N. Engl. J. Med. 294 (10), 526–531. 10.1056/NEJM197603042941005 765821

[B15] Levey A. S. Stevens L. A. Schmid C. H. Zhang Y. Castro A. F. 3rd Feldman H. I. (2009). A New Equation to Estimate Glomerular Filtration Rate. Ann. Intern. Med. 150 (9), 604–612. 10.7326/0003-4819-150-9-200905050-00006 19414839 PMC2763564

[B16] Lyons O. Whelan B. Bennett K. O'Riordan D. Silke B. (2010). Serum Albumin as an Outcome Predictor in Hospital Emergency Medical Admissions. Eur. J. Intern. Med. 21 (1), 17–20. 10.1016/j.ejim.2009.10.010 20122607

[B17] Maclouf J. Kindahl H. Granström E. Samuelsson B. (1980). Interactions of Prostaglandin H2 and Thromboxane A2 with Human Serum Albumin. Eur. J. Biochem. 109 (2), 561–566. 10.1111/j.1432-1033.1980.tb04828.x 7408901

[B18] Marlow N. M. Slate E. H. Bandyopadhyay D. Fernandes J. K. Salinas C. F. (2011). An Evaluation of Serum Albumin, Root Caries, and Other Covariates in Gullah African Americans with Type-2 Diabetes. Community Dent Oral Epidemiol. 39 (2), 186–192. 10.1111/j.1600-0528.2010.00586.x 21070320 PMC3071686

[B19] Mora S. Manson J. E. (2016). Aspirin for Primary Prevention of Atherosclerotic Cardiovascular Disease. JAMA Intern. Med. 176 (8), 1195–1204. 10.1001/jamainternmed.2016.2648 27322595

[B20] Morel O. Jesel L. Abbas M. Morel N. (2013). Prothrombotic Changes in Diabetes Mellitus. Semin. Thromb. Hemost. 39 (5), 477–488. 10.1055/s-0033-1343888 23629823

[B21] Pignatelli P. Farcomeni A. Menichelli D. Pastori D. Violi F. (2020). Serum Albumin and Risk of Cardiovascular Events in Primary and Secondary Prevention: a Systematic Review of Observational Studies and Bayesian Meta-Regression Analysis. Intern. Emerg. Med. 15 (1), 135–143. 10.1007/s11739-019-02204-2 31605272

[B22] Porcellati S. Gresele P. Stasi M. Buratta S. Horrocks L. A. De Franceschi S. (1995). Original Article: Albumin Prevents TxB, Formation from Thrombin-Stimulated Human Platelets by Sequestering the Liberated Arachidonic Acid in the Extracellular Space. Platelets. 6 (6), 381–387. 10.3109/09537109509078476 21043769

[B23] Rocca B. Santilli F. Pitocco D. Mucci L. Petrucci G. Vitacolonna E. (2012). The Recovery of Platelet Cyclooxygenase Activity Explains Interindividual Variability in Responsiveness to Low-Dose Aspirin in Patients with and without Diabetes. J. Thromb. Haemost. 10 (7), 1220–1230. 10.1111/j.1538-7836.2012.04723.x 22471290

[B24] Ronit A. Kirkegaard-Klitbo D. M. Dohlmann T. L. Lundgren J. Sabin C. A. Phillips A. N. (2020). Plasma Albumin and Incident Cardiovascular Disease. Arterioscler Thromb Vasc Biol. 40 (2), 473–482. 10.1161/ATVBAHA.119.313681 31852221

[B25] Santilli F. Pignatelli P. Violi F. Davì G. (2015). Aspirin for Primary Prevention in Diabetes Mellitus: From the Calculation of Cardiovascular Risk and Risk/benefit Profile to Personalised Treatment. Thromb. Haemost. 114 (5), 876–882. 10.1160/TH15-03-0202 26245672

[B26] Santos-Gallego C. G. Badimon J. J. (2014). The Sum of Two Evils. J. Am. Coll. Cardiol. 64, 1926–1928. 10.1016/j.jacc.2014.08.023 25444148

[B27] Santos-Gallego C. G. Badimon J. (2021). Overview of Aspirin and Platelet Biology. Am. J. Cardiol. 144 (Suppl. 1), S2–S9. 10.1016/j.amjcard.2020.12.018 33706986

[B28] Shiyovich A. Sasson L. Lev E. Solodky A. Kornowski R. Perl L. (2020). Relation of Hypoalbuminemia to Response to Aspirin in Patients with Stable Coronary Artery Disease. Am. J. Cardiol. 125 (3), 303–308. 10.1016/j.amjcard.2019.10.055 31787248

[B29] Shoelson S. E. Lee J. Goldfine A. B. (2006). Inflammation and Insulin Resistance. J. Clin. Invest. 116 (7), 1793–1801. 10.1172/JCI29069 16823477 PMC1483173

[B30] Thygesen K. Alpert J. S. White H. D. au fnm. Jaffe A. S. Apple F. S. (2007). Universal Definition of Myocardial Infarction. Circulation. 116 (22), 2634–2653. 10.1161/CIRCULATIONAHA.107.187397 17951284

[B31] Warner T. D. Vojnovic I. Bishop‐Bailey D. Mitchell J. A. (2006). Influence of Plasma Protein on the Potencies of Inhibitors of Cyclooxygenase‐1 and ‐2. FASEB j. 20 (3), 542–544. 10.1096/fj.05-4434fje 16403783

[B32] Wright A. K. Suarez-Ortegon M. F. Read S. H. Kontopantelis E. Buchan I. Emsley R. (2020). Risk Factor Control and Cardiovascular Event Risk in People with Type 2 Diabetes in Primary and Secondary Prevention Settings. Circulation. 142 (20), 1925–1936. 10.1161/CIRCULATIONAHA.120.046783 33196309 PMC7664968

[B33] Zheng S. L. Roddick A. J. (2019). Association of Aspirin Use for Primary Prevention with Cardiovascular Events and Bleeding Events. Jama. 321 (3), 277–287. 10.1001/jama.2018.20578 30667501 PMC6439678

